# Effect of Elevated Temperature on Mechanical Properties of High-Volume Fly Ash-Based Geopolymer Concrete, Mortar and Paste Cured at Room Temperature

**DOI:** 10.3390/polym13091473

**Published:** 2021-05-02

**Authors:** Jun Zhao, Kang Wang, Shuaibin Wang, Zike Wang, Zhaohui Yang, Eskinder Desta Shumuye, Xinglong Gong

**Affiliations:** 1 School of Civil Engineering, Zhengzhou University, Zhengzhou 450001, China; zhaoj@zzu.edu.cn (J.Z.); wkang@gs.zzu.edu.cn (K.W.); wangshuaibin@gs.zzu.edu.cn (S.W.); eskdes@gs.zzu.edu.cn (E.D.S.); 2 School of Mechanics and Safety Engineering, Zhengzhou University, Zhengzhou 450001, China; gongxl@ustc.edu.cn; 3 School of Mechanical and Materials Engineering, Washington State University, Pullman, WA 99164, USA; zhaohui.yang@wsu.edu; 4 CAS Key Laboratory of Mechanical Behavior and Design of Materials, Department of Modern Mechanics, University of Science and Technology of China, Hefei 230027, China

**Keywords:** geopolymer, fly ash and slag blend, elevated temperature exposure, mechanical properties, micro-structure

## Abstract

This paper presents results from experimental work on mechanical properties of geopolymer concrete, mortar and paste prepared using fly ash and blended slag. Compressive strength, splitting tensile strength and flexural strength tests were conducted on large sets of geopolymer and ordinary concrete, mortar and paste after exposure to elevated temperatures. From Thermogravimetric analyzer (TGA), X-ray diffraction (XRD), Scanning electron microscope (SEM) test results, the geopolymer exhibits excellent resistance to elevated temperature. Compressive strengths of C30, C40 and C50 geopolymer concrete, mortar and paste show incremental improvement then followed by a gradual reduction, and finally reach a relatively consistent value with an increase in exposure temperature. The higher slag content in the geopolymer reduces residual strength and the lower exposure temperature corresponding to peak residual strength. Resistance to elevated temperature of C40 geopolymer concrete, mortar and paste is better than that of ordinary concrete, mortar and paste at the same grade. XRD, TGA and SEM analysis suggests that the heat resistance of C–S–H produced using slag is lower than that of sulphoaluminate gel (quartz and mullite, etc.) produced using fly ash. This facilitates degradation of C30, C40 and C50 geopolymer after exposure to elevated temperatures.

## 1. Introduction

Concrete is by far the most widely used construction material today. The most commonly used binder material for conventional concrete is ordinary Portland cement (OPC) [1]. According to the latest statistics from the International Energy Agency, global cement production reached 4.1 billion tons annually in the years of 2013–2019 [2]. As is known, cement manufacturing requires large fuel combustion, as well as decomposition of limestone [1,3]. In addition, it has been reported that cement production emits equivalently one ton of CO_2_ per ton of product, and the ordinary Portland cement industry contributes 5–7% of total worldwide CO_2_ emissions [4,5,6]. To reduce the hazards of the cement industry to the environment, the geopolymer, a new inorganic alumino-silicate polymer, has been developed and is expected to replace OPC in concrete production [1,7,8]. The geopolymer was synthesized from materials of geological origin or by-product materials, such as metakaolin [9,10] blast furnace slag [11] and fly ash [10,12] that are rich in silicon and aluminum using alkali activation. In addition to a minimal impact of the production process on the environment, geopolymer concrete also possesses excellent properties, such as good resistance against acid and sulphate attack, high early age strength, high or low setting time, low shrinkage, and fire resistance and low thermal conductivity [4,13]. Moreover, considering that the concrete structure is likely to be exposed to fire and an elevated temperature environment during the service stage, performance evaluation of geopolymer concrete after exposure to elevated temperature is important to determine its suitability for engineering applications [14,15,16].

Every year, coal-fired power plants worldwide discharge a large amount of fly ash as by-products, and fly ash has become one of the most widely available source materials for use as a geopolymer binder [12,17,18]. Mechanical property evaluation for geopolymer concrete, mortar and paste has shown that the elevated temperature resistance of fly ash-based (generally refers to low-calcium, i.e., Class F fly ash) geopolymer is significantly better than that of slag or metakaolin based geopolymer [9,14,16,19,20,21,22]. However, a disadvantage of fly ash-based geopolymer is that efficient curing must be conducted at a relatively high temperature (e.g., above 60 °C) due to the poor hydration reactivity of fly ash. The heat curing process leads to high costs and energy consumption creates a barrier for the wide application of fly ash-based geopolymer in building processes as the formulation of cast-in-situ concrete [9,12,21,23,24,25,26].

Recently, it has been shown that curing of a fly ash-based geopolymer at ambient temperature could be greatly accelerated after addition of a small proportion of slag [26,27,28,29,30] or OPC [25], with high CaO content. It was concluded that the additional CaO resulted in the formation of hydrated products such as calcium silicate hydrates (C–S–H), along with an alumino-silicate geopolymer network [26,27]. Consequently, a higher content of slag/OPC leads to improved mechanical properties, shorter setting time and a lower slump/flow value for the geopolymer [25,26,27,29]. Further, the optimal slag proportion of fly ash and slag blended into geopolymer concrete cured at ambient temperature is suggested to be in range of 20% to 30% as determined using performance criteria of compressive strength, workability and setting time [30].

It has been found that a fly ash and slag blend-based geopolymer exhibits better elevated temperature resistance than cement-based materials [20]. As the exposure temperature increases, the strength of fly ash and slag blend-based geopolymer displays an initial increase followed by a reduction [31]. Moreover, material containing higher content of slag is less resistant to elevated temperature [20,31,32], indicating that the products from fly ash may have better thermal stability than those from slag. Similarly, the fly ash and slag blend-based geopolymer mortar cured in 70 °C condition was also proved to possess the similar properties with the change of slag content after high temperatures [33]. Therefore, a geopolymer containing a high volume of fly ash may be well-suited for applications requiring elevated temperature resistance. However, most previous work is focused on fly ash and slag blend-based geopolymer paste cured at room temperature. The properties of high-volume fly ash-based geopolymer concrete and mortar cured at ambient temperature has not been fully explored. To promote the application of geopolymer, a knowledge of the mechanical properties of high-volume fly ash-based geopolymer concrete, mortar and paste is necessary.

An experimental study of the mechanical, mineralogical and micro-structural properties of high-volume fly ash-based geopolymer concrete, mortar and paste after exposure to elevated temperature has been conducted. By varying the proportions of slag and fly ash, geopolymer concrete, mortar and paste specimens with three different grades (C30, C40 and C50) were prepared and cured at room temperature. The compressive strength, splitting tensile strength and flexural strength were determined for concrete specimens, and compressive strength for mortar and paste specimens after exposure to elevated temperatures. The effect of slag content on the elevated temperature resistance of geopolymer concrete, mortar and paste and the susceptibility of geopolymer concrete compressive strength, splitting tensile strength and flexural strength to heating temperature was also examined. Moreover, thermogravimetric analysis (TGA), X-ray diffractometry (XRD) and scanning electron microscope (SEM) were conducted to investigate the mass loss, component transformation and micro-structural changes of geopolymer paste, respectively.

## 2. Experimental Program

### 2.1. Materials

**Figure 1:**
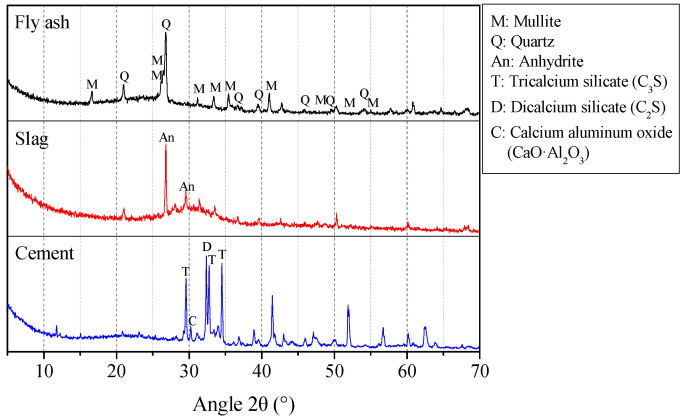
XRD result of fly ash, slag and cement.

In this study, Class F fly ash and ground granulated blast-furnace slag were used as the geopolymer binder materials. The ordinary Portland cement (OPC) with type PO 42.5 was used to prepare OPC binder, which was also referred as ordinary binder in this paper. Finesses of cement using 80 μm sieve was 99.1%, whereas, for fly ash and slag recorded as 91.1% and 94.0% using 45 μm sieve respectively [34]. The chemical compositions determined by X-Ray Fluorescence (XRF, model: Rigaku ZSX primus, Rigaku Corporation, Tokyo, Japan) of fly ash, slag and OPC are shown in Table 1. Further, the XRD (model: Bruker D8, Bruker Corporation, Billerica, America) patterns are presented in Figure 1. The fly ash contained peaks corresponding to unreactive crystalline phases quartz (SiO_2_) and mullite (e.g., Al_6_Si_2_O_13_ [31]). The XRD pattern of the slag showed peaks corresponding to anhydrite (CaSO_4_) and a diffuse band at 25–35° 2θ associated with the presence of a glassy phase [31,32]. The major peaks of OPC correspond to tricalcium silicate (C_3_S), dicalcium silicate (C_2_S) and calcium aluminum oxide (CaO Al_2_O_3_) [35].

**Figure 2:**
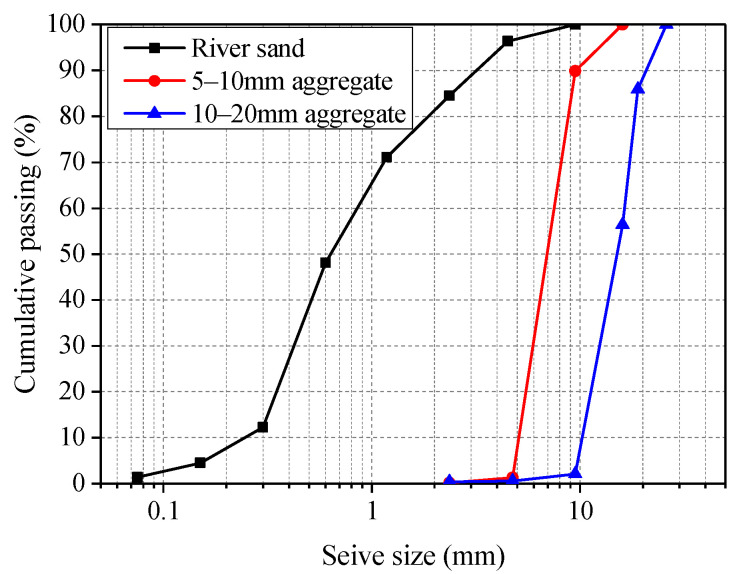
Particle size distribution curve of river sand and coarse aggregate.

The alkaline activator of geopolymer binder was a mixture of sodium hydroxide (NaOH) and sodium silicate (Na_2_O·*n*SiO_2_) solutions. The NaOH solution of 14 M concentration was prepared by mixing 98.7% pure pallets with tap water. The mole ratio of SiO_2_ to Na_2_O of the sodium silicate solution was 2.73 with chemical compositions of 30.95% SiO_2_, 11.69% Na_2_O and 57.36% water. The fine aggregate was natural river sand with fineness modulus of 2.71, and moisture content of 3.09%. Coarse aggregates were a combination of crushed gravel with grain sizes of 5–10 mm and 10–20 mm in a mass ratio of 3:7. The particle size distribution for fine and coarse aggregates is shown in Figure 2. Normal tap water was used during concrete mixing.

### 2.2. Specimen Preparation

Based on previous reports [27,36], geopolymer concrete, mortar and paste specimens in three different grades (C30, C40 and C50) were prepared. Grades of the geopolymer specimens were controlled by changing the proportion of slag and fly ash. During the concrete mixing, a specific amount of water was introduced to improve the workability of geopolymer mixture. OPC concrete, mortar and paste specimens with grade of C40 were also prepared as a reference. The detailed mix proportions of all concrete, mortar and paste in this study are listed in Table 2.

Mixing geopolymer specimens contains two steps: preparation of alkaline activator and followed by mixing of all ingredients. The alkaline activator was prepared by mixing sodium silicate and sodium hydroxide solutions with a ratio of 2.5:1 about 48 h before final mixing with the remaining ingredients. The mixing sequence of geopolymer concrete was as follows: fly ash, slag, and fine aggregate were first mixed together for two minutes, coarse aggregate was then added and dry-mixed for additional two minutes, and alkaline activator and water were finally introduced and mixed for four more minutes. A similar mixing sequence of geopolymer concrete was followed for geopolymer mortar and paste apart from the removal of coarse aggregate, and both coarse and fine aggregate, respectively. Similarly, the mixing procedure of ordinary concrete, mortar and paste specimens were the same to that of the geopolymer specimens except replacing the binder materials (fly ash and slag) and alkaline activator with OPC and water, respectively. After mixing, the fresh concrete, mortar and paste mixtures were cast into various molds (cube with size of 150 mm for compressive and splitting tensile tests of concrete, prism with size of 100 mm × 100 mm × 400 mm for flexural tensile strength test of concrete, and cube with size of 70.7 mm for compressive test of mortar and paste) in two layers and each layer was compacted on a vibrating table. All the specimens were then cured at ambient temperature with plastic film covering. After 24 h, the specimens were de-molded and placed in a standard curing chamber (20 ± 2 °C, not less than 95% RH) [37] for curing until the day of testing.

### 2.3. Heating System

**Figure 3:**
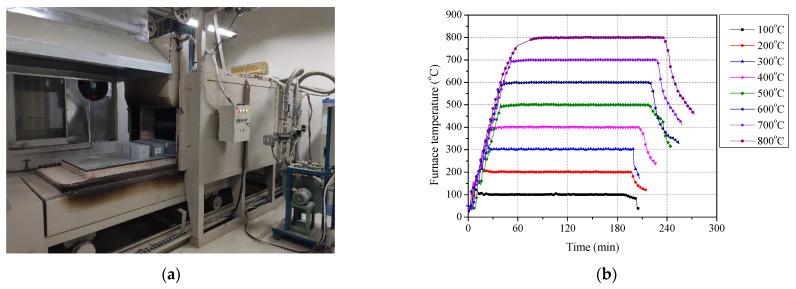
Heating system for concrete, mortar and paste: (**a**) furnace and (**b**) measured furnace temperature-time curves.

At 28 days of curing, the concrete, mortar and paste specimens were heated using a gas-electric hybrid furnace (Changsha Kehui Furnace Technology Co., Ltd., Changsha, China) controlled by a computer program, as shown in Figure 3a, the inner dimension of which is 2200 mm × 700 mm × 600 mm. All the specimens were kept for 3 h in each of the target temperatures, in range of 100 °C to 800 °C with an interval of 100 °C, and the heating rate of furnace was 15 °C/min. After heating, the door of furnace was opened, and then the specimens were left to cool in air and ready for the further test. The measured temperature curves of furnace are shown in Figure 3b. In addition, for unheated specimens (unexposed specimens), the exposure temperature was recorded as 25 °C, which refers to room temperature.

### 2.4. Mass Loss Test

The mass losses of exposed concrete, mortar and paste specimens were tested before the mechanical test. The mass loss was determined by weighting the exposed specimens before and after heating. The size of concrete specimen was 150 mm cube and that of mortar and paste specimens was 70.7 mm cube. At least three specimens were tested for each exposure condition.

### 2.5. Mechanical Property Test

The mechanical property tests of unexposed specimens (at curing ages of 3, 7, 28 and 56 days) and exposed specimens were carried out according to modified GB 50081-2019 [37] and JGJ/T 70-2009 [38] for concrete and for mortar and paste, respectively. The compressive strength of concrete (150 mm cube) was tested at a loading rate of 0.5 MPa/s by using Hydraulic universal testing machine (model: YES-3000, Beijing road industry Keyu Test Instrument Co., Ltd., Beijing, China) with the capacity of 3000 kN. The splitting tensile strength of concrete (150 mm cube) was tested at a loading rate of 0.05 MPa/s by using Hydraulic universal testing machine (model: WEW-600D, Jinan Hengle Xingke Instrument Co., Ltd., Jinan, China) with the capacity of 600 kN. The flexural strength of concrete (100 mm × 100 mm × 400 mm prism) was measured by the four-point bending test method at a loading rate of 0.05 MPa/s using an electronic universal testing machine with the capacity of 300 kN. In contrast, the compressive strength of mortar and paste specimens (70.7 mm cube) were tested at a loading rate of 0.2 MPa/s with a hydraulic universal testing machine with the capacity of 600 kN. Each test was conducted for at least three times.

### 2.6. Thermogravimetric Analysis/Differential Scanning Calorimetry (TGA/DSC)

In this study, thermogravimetric analysis (TGA) was conducted for unexposed geopolymer (with grade of C30, C40, C50) and OPC (with grade of C40) paste at 28 days of curing age for characterization of water content of binder [39]. The test specimens for TGA, obtained from the residual fragments of paste specimen after compression test, were dried at 60 °C for 24 h prior to TGA analyses. The ground paste was subjected to TGA and differential scanning calorimetry (DSC) using a simultaneous TGA/DSC thermal analyzer (model: NETZSCH STA 409 PC, NETZSCH Scientific Instruments Trading Ltd., Selbu, Germany) with a heating rate of 10 °C/min from 30 to 900 °C temperature range.

### 2.7. X-ray Diffraction (XRD)

The X-ray diffraction (XRD) patterns of raw binder materials (i.e., fly ash, and slag cement) and unexposed and exposed geopolymer and ordinary paste were obtained using CuKα radiation (λ = 1.5418 Å) at 40 kV and 30 mA. Similar to specimen’s preparation for TGA, the specimens for XRD were also taken from the residual fragments of paste compression specimens, dried at 60 °C for 24 h and ground before running the XRD test. Scanned 2θ angle from 5° to 80° was adopted with step size of 0.0334° in this study.

### 2.8. Scanning Electron Microscope (SEM)

The morphology and micro-structures of unexposed and exposed geopolymer and ordinary paste were observed with a Zeiss/Auriga FIB scanning electron microscope (SEM, Carl Zeiss AG, Oberkochen, Germany). Before SEM test, the specimens, which were also chosen from the residual fragments of paste compression specimen dried in 60 °C for 24 h, was coated with gold to make a conductive surface.

### 2.9. Definition of Specimen Label

In this study, the specimen labels for all the tested concrete, mortar and paste were named as follows:

Geopolymer and OPC (or ordinary) are denoted by the letters “G” and “O”, respectively. Concrete, mortar and paste are denoted by the letters “C”, “M” and “P”, respectively. In the label “GC-C30”, ‘‘G” means geopolymer, the first letter “C” means concrete, and “C30” means specimen with grade of C30, i.e., the compressive strength of specimen is 30 MPa. Similarly, “OM-C40” represents the ordinary mortar specimen with grade of C40, i.e., compressive strength of 40 MPa; “OP-C50” represents the ordinary paste specimen with grade of C50, i.e., compressive strength of 50 MPa.

## 3. Results and Discussion

### 3.1. Basic Mechanical Properties of Unexposed Specimens

**Figure 4:**
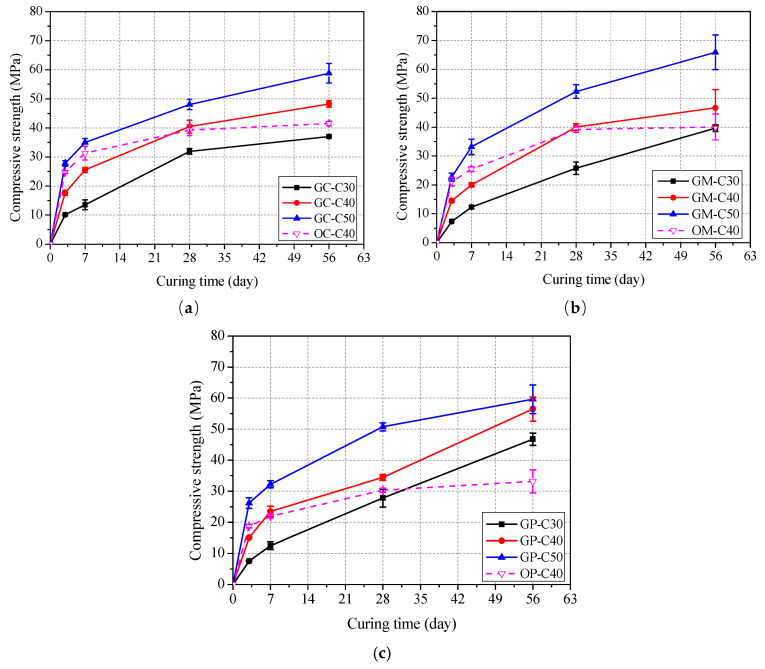
Change of compressive strength of cube specimen with curing time: (**a**) concrete; (**b**) mortar and (**c**) paste.

The development of compressive strength of unexposed concrete, mortar and paste with curing time at room temperature are shown in Figure 4 and Table 3. It is clear that the compressive strengths of geopolymer concrete, mortar and paste are all effectively improved with increase of slag content in the binder (see in Table 3). Similar experimental results and detailed improvement mechanism of slag in fly ash based geopolymer can be found in previous literatures [26,27,30,40]. Figure 4 also show that the compressive strength development trend of geopolymer specimens is obviously different from that of ordinary specimens. For geopolymer and ordinary concrete, mortar and paste specimens with grade of C40, the growth rate of strength of geopolymer specimen is lower than that of ordinary specimen in the first 7 days. After 7 days, the growth rate of geopolymer specimen is higher than that of ordinary specimens, and especially after 28 days, the strength of ordinary specimens increases quite slowly, while that of geopolymer specimens continues to increase significantly. The 28-day compressive strengths of GC-C30, GC-C40, GC-C50 and OC-C40 are 31.9, 40.4, 48.0 and 39.2 MPa, respectively. Meanwhile, the 28-day compressive strengths of GM-C30, GM-C40, GM-C50 and GM-C40 are 25.8, 40.0, 52.3 and 39.2 MPa, respectively, and those of GP-C30, GP-C40, GP-C50 and OP-C40 are 27.8, 34.4, 50.7 and 30.3 MPa, respectively. This shows that concrete, mortar and paste specimens basically reach the target strength levels except for two groups (i.e., 25.8 MPa for GM-C30, and 30.3 MPa for OP-C40). It is noted that the low compressive strength of OP-C40 specimen may be related to the high water to cement ratio.

### 3.2. Surface Morphology and Mass Loss

**Figure 5:**
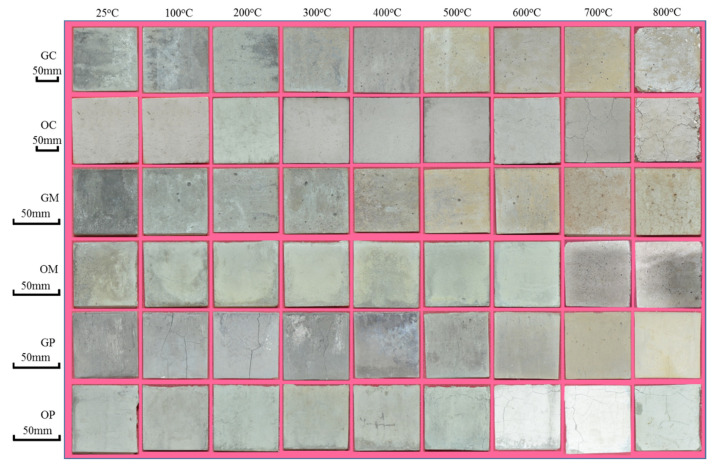
Change of surface morphology of geopolymer and ordinary concrete, mortar and paste (all specimens with grade of C40).

**Figure 6:**
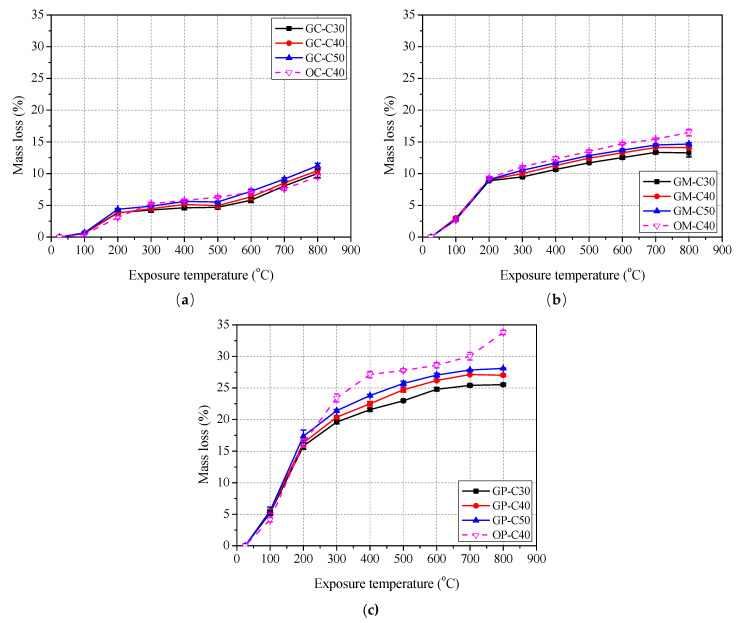
Mass loss rate of concrete, mortar and paste after exposure to elevated temperature: (**a**) concrete; (**b**) mortar and (**c**) paste.

Figure 5 and Figure 6 shows the surface morphology and mass losses of concrete, mortar and paste specimens with different exposure temperatures.

As shown in Figure 5, the changes of surface morphology were similar for GC and GM specimens. No visible cracks were found below 500 °C, then visible cracks started to appear and became wider and denser gradually in the range from 500 to 700 °C, and finally cracks expanded and linked up and specimens became loose above 700 °C. Compared with GC and GM specimens, OC and OM specimens appeared visible cracks at lower exposure temperature (at 300 °C), and the surface damage is more serious at 800 °C. Different from GC and GM specimens, there were obvious cracks on the surface of the GP specimens below 300 °C, then the cracks tended to decrease in the range from 300 to 600 °C, and finally the cracks became denser again above 600 °C. The reasons for this phenomenon need to be further explored. Similarly, the cracks gradually increased with increase in exposure temperature for OP specimens, which lead to more cracks than GP specimens above 600 °C.

As shown in Figure 6, the mass losses of geopolymer and ordinary concrete, mortar and paste specimens increased gradually as the exposure temperature goes up. For geopolymer concrete, the mass losses of specimens increased rapidly below 200 °C, then increased slowly in the range from 200 to 500 °C, and then increased rapidly again above 500 °C. For geopolymer mortar and paste, the mass losses of specimens increased rapidly below 200 °C, and then increased slowly and tended to be stable in the range from 200 to 800 °C. A remarkable phenomenon was found for geopolymer concrete, mortar and specimens, that is, the higher strength grade leaded to the higher mass losses, especially when the exposure temperature exceeded 300 °C. This is because that the mass losses of geopolymer specimens at the lower temperatures (i.e., 100 and 200 °C) is mainly attributed to evaporation of water, and the water contents of C30, C40 and C50 geopolymer formulas are same (see in Table 2). At the higher temperatures (above 300 °C), the mass loss of geopolymer additionally includes the decomposition of internal components, which reveals that the product of slag reaction is more easily decomposed than that of fly ash at higher temperature (above 300 °C). The higher mass losses may cause the worse thermal stability for geopolymer specimens, which was verified by the subsequent mechanical properties results. For the geopolymer specimens with the same strength grade, the water content of concrete, mortar and paste increased in turn, which is not strange due to the minimal moisture contained in coarse and fine aggregates. In addition, Figure 6a shows the mass loss of OC-C40 is higher than that of GC-C40 in the range of 300 to 600 °C, but lower than that of GC-C40 at 700 and 800 °C. Figure 6b,c shows that the mass losses of OM-C40 and OP-C40 are higher than that of GM-C40 and GP-C40 in the range from 300 to 800 °C, respectively. This basically indicates that geopolymer processes better thermal stability than ordinary specimens.

### 3.3. Residual Mechanical Properties of Exposed Specimens

#### 3.3.1. Concrete

**Figure 7:**
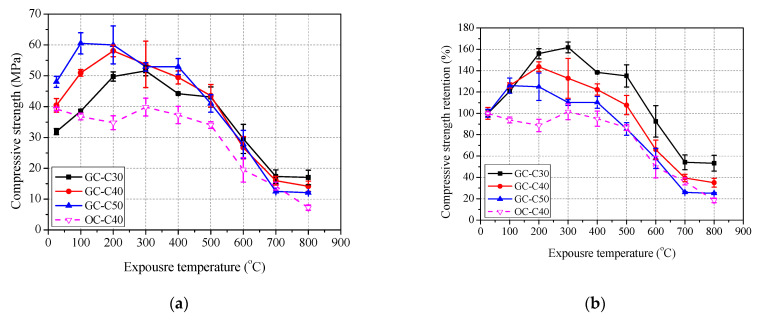
Residual compressive strength of concrete after exposure to elevated temperature: (**a**) compressive strength and (**b**) retention.

**Figure 8:**
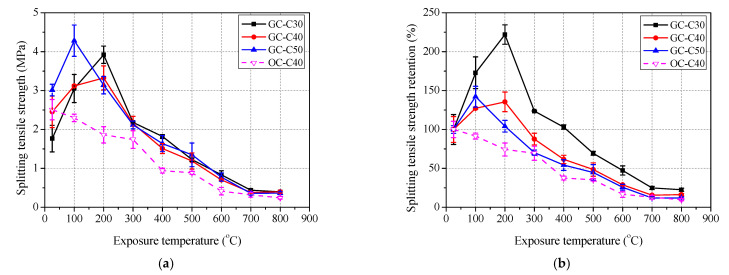
Residual splitting tensile strength of concrete after exposure to elevated temperature: (**a**) splitting tensile strength and (**b**) retention.

**Figure 9:**
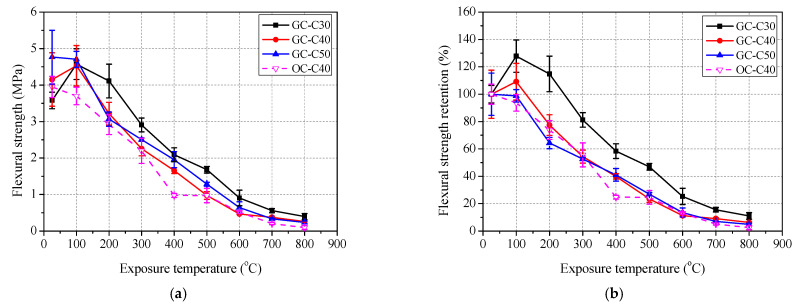
Residual flexural strength of concrete after exposure to elevated temperature: (**a**) flexural strength and (**b**) retention.

The variation of residual mechanical properties of concrete after exposure to elevated temperatures are shown in Figure 7, Figure 8 and Figure 9, and detailed data are summarized in Table 4.

As shown in Figure 7a, the compressive strength of geopolymer concrete increased first and then deceased gradually, and finally basically remained unchanged or even increased slightly with the increase of exposure temperature. The improvement of compressive strength was generally attributed to the sintering reactions of unreacted fly ash at elevated temperature forming a more compact microstructure [9,16,20]. Figure 7b shows that the peak residual compressive strength retention is 161.7% at 300 °C, 143.6% at 200 °C and 126.0% at 100 °C for GC-C30, GC-C40 and GC-C50, respectively. Further, after exposure to 800 °C, the residual compressive strength retention of GC-C30, GC-C40 and GC-C50 is 53.3%, 35.1% and 25.2%, respectively. Clearly, the higher content of slag of concrete caused the lower residual compressive strength and the lower exposure temperature corresponding to peak residual compressive strength. This revealed that GC-C30 achieves the best elevated temperature resistance, followed by GC-C40 and GC-C50. Figure 7 also shows that the compressive strength of OC-C40 decreases slightly below 200 °C, and then returns to the initial level at 300 °C, and gradually decreased above 300 °C, and the reduction rate obviously increase above 500 °C. Similar test results of ordinary Portland cement- based materials and detail reaction mechanism were reported in [41,42,43]. After 800 °C exposure, the residual compressive strength retention of OC-C40 is only 18.4%. Figure 7b shows that the residual compressive strength of GC-C40 is obviously higher than that of OC-C40 at each exposure temperature. This proves the geopolymer concrete has attained better elevated temperature resistant than ordinary concrete.

As shown in Figure 8 and Figure 9, the variation of splitting tensile strength flexural strength of geopolymer concrete with exposure temperature is basically close to that of compressive strength. It is found that the peak residual splitting tensile strength retention is 222.0% at 200 °C, 135.5% at 200 °C and 142.1% at 100 °C for GC-C30, GC-C40 and GC-C50, respectively. After exposure to 100 °C, the flexural strength retentions of GC-C30, GC-C40 both reach their respective peaks, which are 127.8% and 109.1%, respectively. One in particular is the flexural strength retention curve of GC-C50, which lacks the rising section and decreases directly with the increase of exposure temperature. After exposure to 800 °C, the residual splitting strength and flexural strength retentions are 22.6% and 11.1% for GC-C30, 16.3% and 6.3% for GC-C40, and 12.1% and 4.9% for GC-C50, respectively. Clearly, the higher content of slag in geopolymer concrete also leads to the lower residual splitting and flexural strength retentions. In addition, different from the compressive strength retention curve of OC-C40, the curves of splitting tensile strength and flexural strength retentions of OC-C40 both decreases gradually except for a small plateau in the range of 400 to 500 °C. The residual splitting strength and flexural strength retentions of OC-C40 exposed to 800 °C are 9.7 and 2.4%, respectively. Meanwhile, Figure 8b and Figure 9b) shows that the splitting tensile strength and flexural strength retentions of GC-C40 are basically higher than those of OC-C40 at each exposure temperature, which again shows the more resistant to elevated temperature of geopolymer concrete than ordinary concrete.

**Figure 10:**
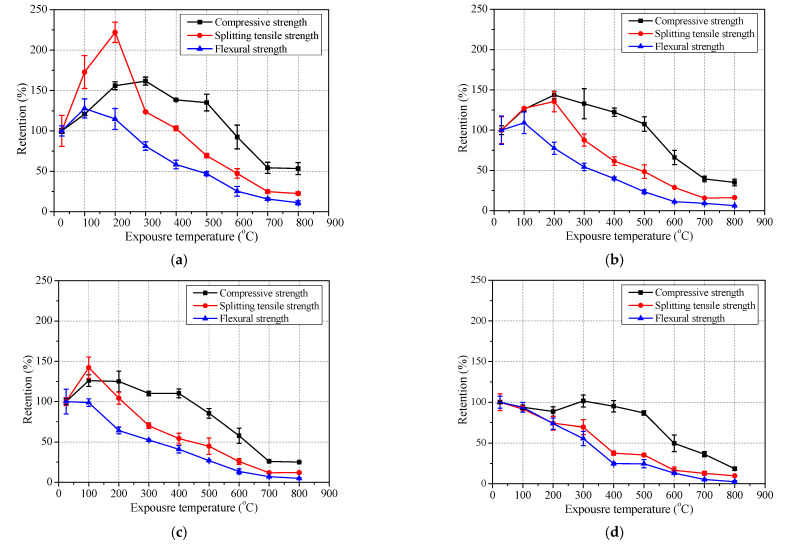
Comparison of compressive strength, splitting tensile strength and flexural strength retention of concrete after exposure to elevated temperature: (**a**) GC-C30; (**b**) CG-C40; (**c**) GC-C50 and (**d**) OC-C40.

The relationship among those which consist of compressive strength, splitting tensile strength and flexural strength of geopolymer concrete and ordinary concrete after exposure to elevated temperatures was compared in Figure 10. It was found that the retention of compressive strength is highest, then the splitting tensile strength, and then the flexural strength is lowest after exposure to elevated temperatures whether with geopolymer concrete or ordinary concrete. Therefore, the susceptibility of flexural strength, splitting tensile strength and compressive strength to elevated temperature decreased in turn for different types of concrete.

#### 3.3.2. Mortar

**Figure 11:**
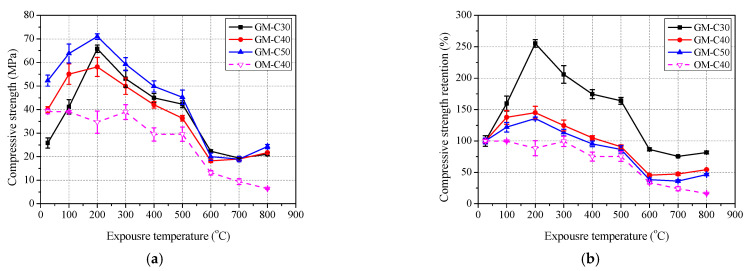
Residual compressive strength of mortar after exposure to elevated temperature: (**a**) compressive strength and (**b**) retention.

The test results of compressive strength of mortar with exposure temperature are shown in Figure 11 and Table 5. It was found that the variation trend of compressive strengths of GM-C30, GM-C40, GM-C50 and OM-C40 are similar with that of compressive strength of GC-C30, GC-C40, GC-C50 and OC-C40, respectively. The compressive strength retentions of GM-C30, GM-C40 and GM-C50 all reached their peaks at 200 °C, and the peaks decreased successively, by 255.2%, 145.1% and 135.8%, respectively. Meanwhile, after exposure to 800 °C, the residual compressive strength retentions of GM-C30, GM-C40, GM-C50 also decreased in turn, by 81.6%, 54.3% and 46.4%, respectively. Obviously, the higher content of slag in geopolymer mortar also caused the higher residual compressive retention. In addition, the residual compressive strength retention of OM-C40 is lower than that of GM-C40 in each exposure temperature and is only 16.3% after exposure to 800 °C. Therefore, the heat resistance of geopolymer mortar is better than that of ordinary mortar.

#### 3.3.3. Paste

**Figure 12:**
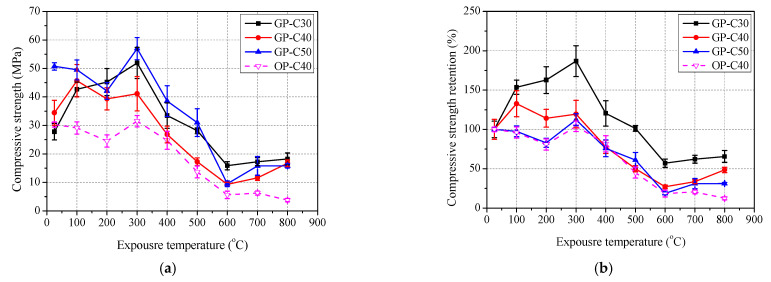
Residual compressive strength of paste after exposure to elevated temperature: (**a**) compressive strength and (**b**) retention.

The test results of compressive strength of paste with exposure temperature are shown in Figure 12 and Table 5. Similarly, the variation trend of compressive strengths of GP-C30, GP-C40, GP-C50 and OM-C40 are basically consistent with that of compressive strength of GC-C30, GC-C40, GC-C50 and OC-C40, respectively. The peak residual compressive strength retentions are 186.9% at 300 °C, 132.6% at 100 °C and 112.3% at 300 °C for GP-C30, GP-C40 and GP-C50, respectively. The residual compressive strength retentions of GP-C30, GP-C40, GP-C50 and OC-C40 are 65.6%, 48.5%, 31.2% and 12.4%, respectively. Clearly, with the increase of slag content, the elevated temperature resistance of geopolymer paste becomes worse, and geopolymer paste is more resistant to elevated temperature than ordinary paste. This agrees with the conclusion of mortar and concrete mentioned above.

#### 3.3.4. Comparison of Concrete, Mortar and Paste

**Figure 13:**
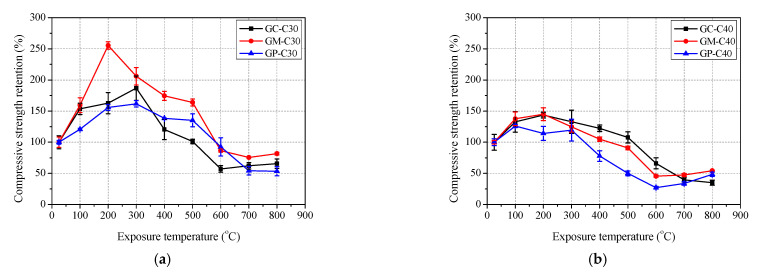

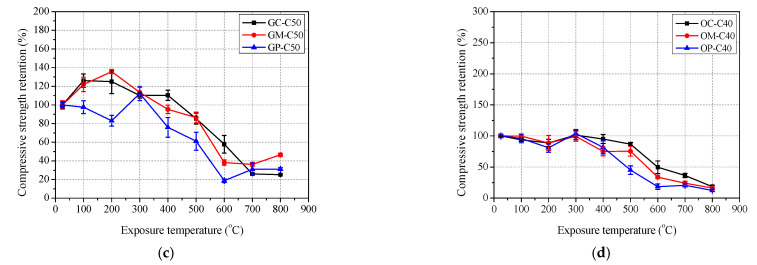
Comparison of compressive strength among concrete, mortar and paste after exposure to elevated temperature: (**a**) geopolymer-C30; (**b**) geopolymer-C40; (**c**) geopolymer-C50 and (**d**) OPC-C40.

The comparison of compressive strength retention curves of concrete, mortar and paste is shown in Figure 13. As in Figure 13a, for C30 geopolymer specimens, the curve of concrete is highest, but those of mortar and paste cross each other. Similarly, the phenomenon of two or three curves crossing each other was also found in C40 and C50 geopolymer and C40 ordinary specimens (Figure 13a–c). Therefore, a defined conclusion on the precise influence of the introduction of sand and coarse aggregate on the elevated temperature resistance of geopolymer and ordinary Portland cement based materials cannot be obtained.

### 3.4. Mineralogical and Micro-Structural Prosperities

Due to the evolution of residual mechanical properties the concrete, mortar and paste with the same cementitious materials was basically consistent and the paste had a higher purity than concrete and mortar. The paste was chosen for the test of mineralogical and micro-structural properties to analyze the morphological and mineralogical change in geopolymer materials after exposure to elevated temperatures.

#### 3.4.1. TGA/DSC Results

**Figure 14:**
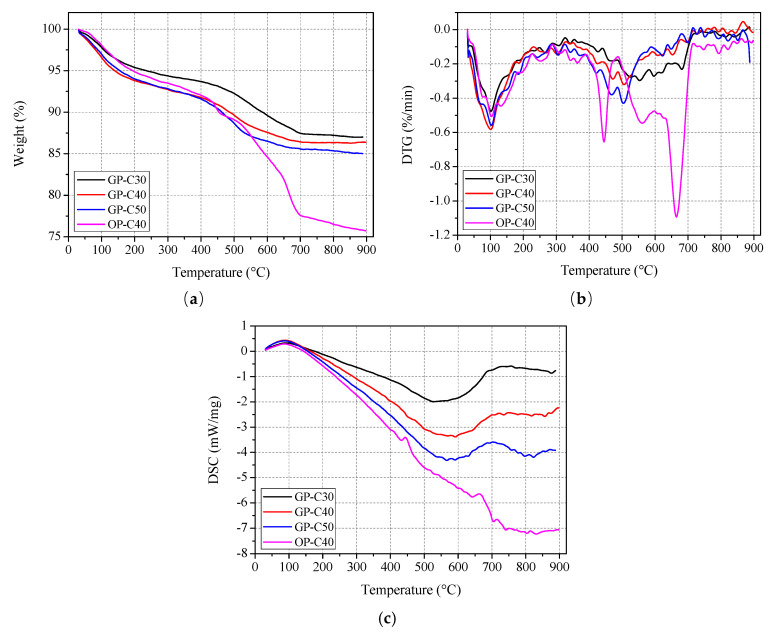
TGA/DSC results: (**a**) weight; (**b**) DTG and (**c**) DSC.

Figure 14 presents the TGA/DSC results of unexposed geopolymer and ordinary paste. As shown in Figure 14a, the weight loss of geopolymer paste initially increased rapidly at 100–200 °C, then slowly at 200–400 °C, then again dramatically at 400–700 °C, and finally basically remained stable at 700–900 °C. With the increase of grade of geopolymer, the weight loss seems to be increased at the same heating temperature, especially above 400 °C. At 800 °C, the weight losses of GP-C30, GP-C40 and GP-C50 are 12.8%, 13.7% and 14.7%, respectively. Considering the same water content of three kinds of geopolymer paste formulations, the higher weight loss of geopolymer paste should be attributed to the higher content of slag. The more slag generally produced more C–S–H in slag and fly ash mixing based geopolymer, which was more easily decomposed at elevated temperature than the production of fly ash. A similar study was given in [39], which showed that the weight loss of fly ash and slag-based polymer paste increases with the increase of slag content. For C40 ordinary paste, the curve of weight loss with heating temperature is similar to that of geopolymer paste. The weight loss of OP-C40 increased rapidly at 100–200 °C, slowly at 200–400 °C, again sharply at 400–700 °C, and finally declined at 700–900 °C. Meanwhile, the weight loss of OP-C40 is lower than that of GP-C40 below 400 °C. However, above 400 °C, the increase of weight loss is obviously accelerated, and the weight loss of OP-C40 is 23.5% at 800 °C, which is higher than 14.7% of GP-C40. Figure 14b further shows the derivative thermogravimetric analysis (DTG) results of paste. It is found that there are two peaks for DTG curves of geopolymer specimens at about 100 and 500–550 C, while four main peaks for that of OP-C40 at about 100, 450, 560 and 665 °C. The weight loss in the peak at about 100 °C generally represents the evaporation of water [22], which are known as the free and weakly absorbed water and reside in pores roughly above 5 nm [44]. The peak of GP-C30 appears at about 550 °C, which is higher that of GP-C40 and GP-C50 (about 500 °C). This also suggested that the higher the slag content, the lower is the decomposition temperature of the product in geopolymer paste. Accordingly, based on previous researches [42,45], for GP-C30 specimen, the weight loss of GP-C30 in the peak at about 445, 560 and 665 °C are mainly due to decomposition/transformation of portlandite (Ca(OH)_2_), C–S–H and calcite (CaCO_3_), respectively. These results again prove that the heat resistance of geopolymer paste is better than ordinary Portland cement paste. Clearly, the TGA results are consist with the mass loss results of bulk paste specimens (Figure 6). In addition, Figure 14c presents the DSC results. Clearly, a broad and flat endothermic peak located from 20 to about 150 °C for all paste specimens, corresponding to the first major weight loss of pate (See in Figure 14a,b), may be ascribed to the loss of free water [46] and the hydrolysis/decomposition of C–S–H and ettringite [47]. Meanwhile, the DSC curves of three geopolymer pastes all show the obvious endothermic peak at 600–900 °C, which is attributed to the crystallization of zeolite (N-A-S-H) and/or zeolite-like substance [46,48], and it is interesting that the temperatures corresponding to the endothermic peaks of GP-C30, GP-C40 and GP-C50 seem to decrease in turn. For OP-C40, in addition to the endothermic peak at 100 °C, there are two more endothermic peaks at about 450 and 650 °C, which were widely reported to be related to the dehydroxylization/decomposition of calcium hydroxide (Ca(OH)_2_) and decarbonation/decomposition of calcite (CaCO_3_), respectively [39,47,49].

#### 3.4.2. XRD Results

**Figure 15:**
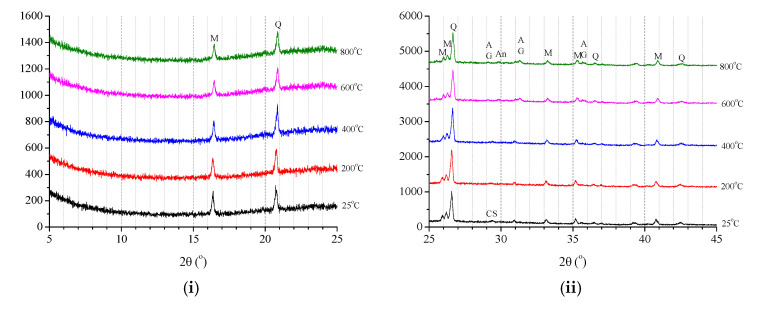

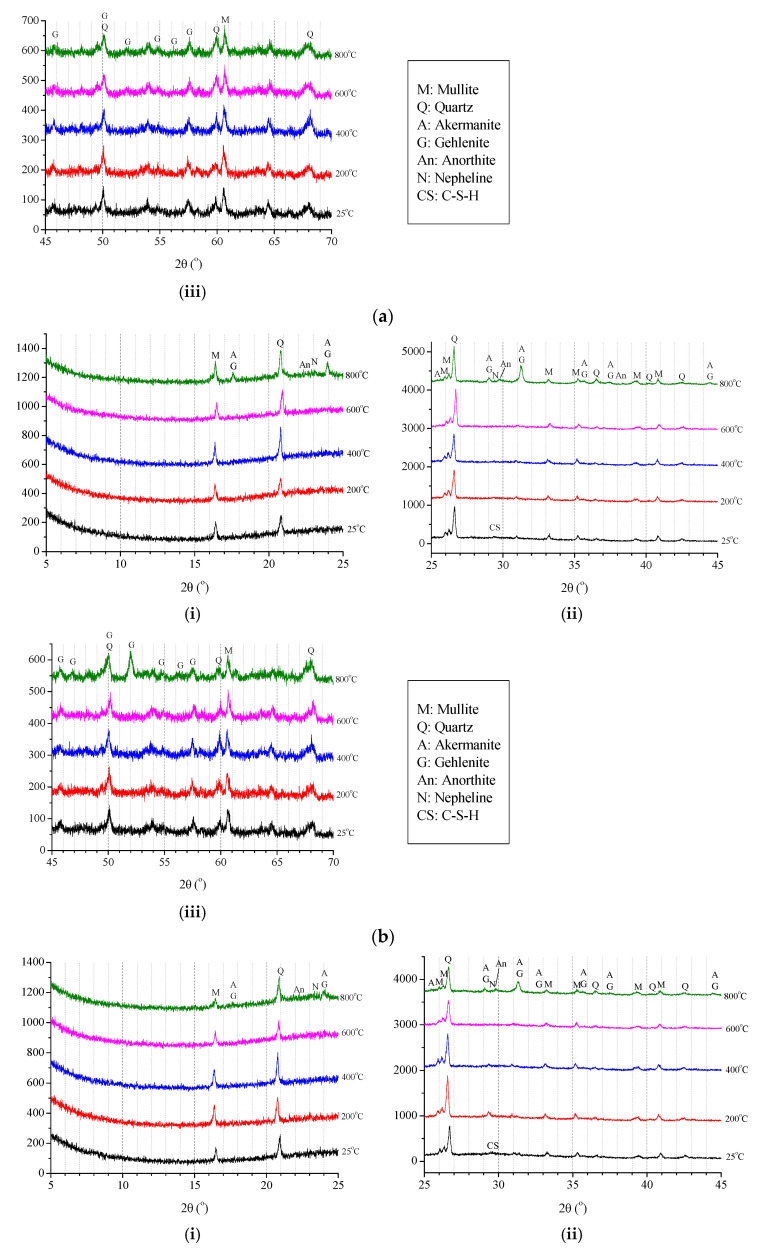

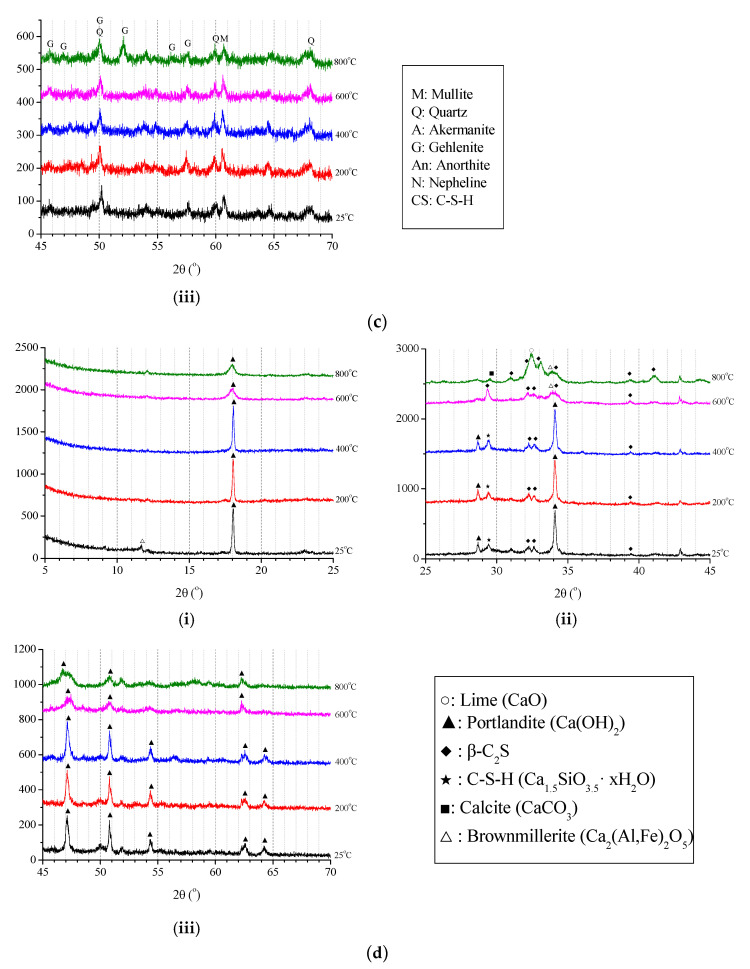
XRD results of geopolymer and ordinary paste after exposure to elevated temperature: (**a**) GP-C30; (**b**) GP-C40; (**c**) GP-C50 and (**d**) OP-C40.3.4.3. SEM Results.

The XRD results of geopolymer and ordinary paste after various elevated temperatures exposure are presented in Figure 15. On the one hand, for geopolymer pastes, Figure 15a–c show that there are no significant differences in the XRD patterns of unexposed GP-C30, GP-C40 and GP-C50, and the variation of XRD patterns of these three kinds of geopolymer paste with exposure temperature are basically the same. This revealed that the reaction products and degradation mechanics of GP-C30, GP-C40 and GP-C50 after elevated temperature exposure are similar, although there are some differences in slag content among the three groups, i.e., 10–30%. Specifically, for the XRD patterns of unexposed geopolymer paste, a diffuse band at 20–28° 2θ associated with the presence of aluminosilicate gel and a diffuse peak at 29–30° 2θ corresponding to C–S–H were observed [50], which suggested that the main reaction products are aluminosilicate gel and C–S–H [31]. Meanwhile, other crystalline phases such as quartz and mullite were also present in the unexposed geopolymer specimens. After exposure to 200 and 400 °C, the XRD patterns of geopolymer paste had little change compared with those at room temperature, which indicated that there was no obvious deterioration of specimen in this condition. However, after exposure to 600 °C, the peak intensity of C–S–H was evidently reduced, indicating that the C–S–H dehydrated [42], while other phases (i.e., aluminosilicate gel, quartz and mullite) still existed. Moreover, the crystalline phases of akermanite and gehlenite, which were contained in the raw slag and disappeared upon the blending with fly ash and alkaline activation, started to reappear in the XRD pattern [32]. Further, the XRD patterns changed significantly after exposure to 800 °C, that is, the peak corresponding to C–S–H disappeared and the peak of akermanite, gehlenite and anorthite was readily apparent. This suggested that C–S–H had fully dehydrated or crystallized, and the main phases in this specimen were akermanite (Ca_2_MgSiO_7_), gehlenite (Ca_2_Al[AlSiO_7_]), anorthite (CaAl_2_Si_2_O_8_), and minor quantity of nepheline ((Na,K)AlSiO_4_) [31]. Clearly, the heat resistance of C–S–H produced by slag is weaker than that of sulphoaluminate gel (quartz and mullite, etc.) produced by fly ash. Therefore, this can explain why the elevated temperature resistance of GP-C30, GP-C40 and GP-C50 decreases in turn. Similar XRD results of fly ash and slag blending geopolymer paste/mortar with slag content of 10–50% [22], 50% [51] and 25–75% [31,32,52] after elevated temperature exposure were also reported respectively by different researchers. Moreover, a hypothesis about the change of phase compositions of fly ash/slag mixed geopolymer paste with exposure temperature was put forward by Park et al. [32].

On the other hand, for ordinary paste, Figure 15d shows that the main phases of unexposed OP-C40 included portlandite (Ca(OH)_2_), C–S–H, brownmillerite and β-C2S, and the XRD patterns of OP-C40 had no significant change after 200 and 400 °C exposure. However, the peak related to C–S–H disappeared and the significant reduction of portlandite significantly decreased after 600 °C exposure, while the intensities of peak related to β-C2S was increased, which is a by-product of C–S–H decomposition [42,53]. After exposure to 800 °C, the intensity of β-C2S was further increased, and the lime (CaO) formed by the Ca(OH)_2_ decomposition was clearly detected as well. The calcite (CaCO_3_) could be also captured at 800 °C. It may be assumed that CaO formed by Ca(OH)_2_ decomposition reacts with CO_2_ present in the furnace and forms CaCO_3_ [41]. These results are basically consistent with the well-known conclusions of ordinary cement-based materials reported by previous researchers [41,42,45,53,54]. On the whole, the XRD results indicated that the thermal stability of geopolymer paste was better than that of cement-based paste. This again agreed with the mechanical properties results.

#### 3.4.3. SEM results

**Figure 16:**
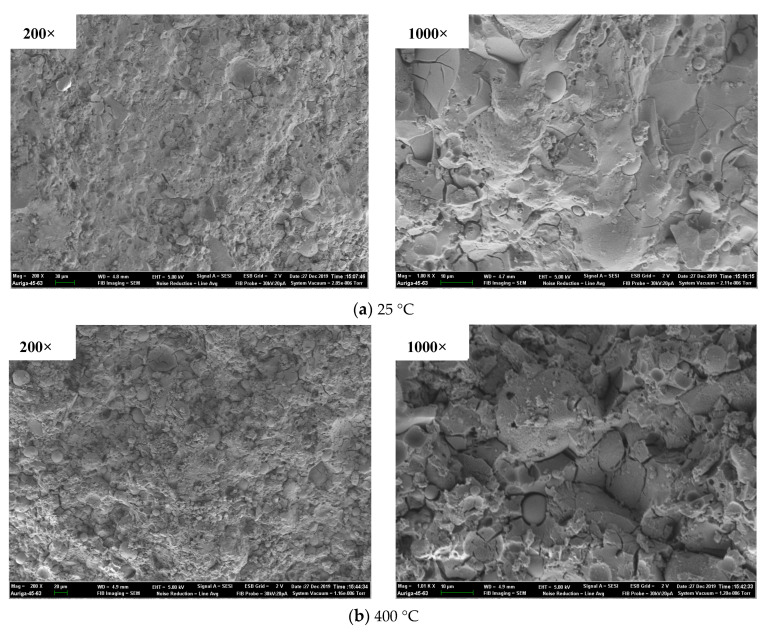

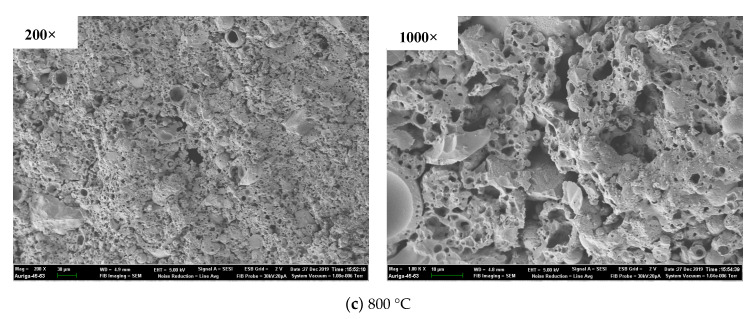
SEM result of GP-C40 after exposure to elevated temperature.

**Figure 17:**
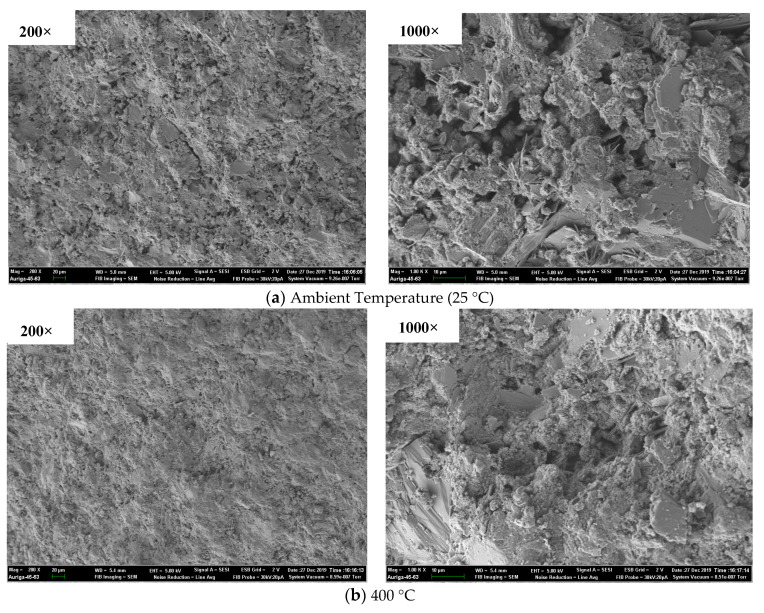

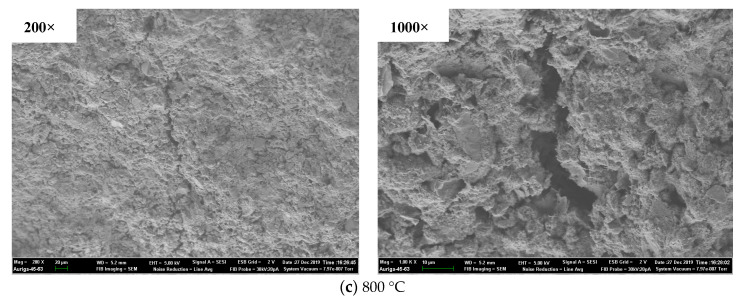
SEM result of OP-C40 after exposure to elevated temperature.

The SEM results of geopolymer and ordinary paste are shown in Figure 16 and Figure 17. It was noted that only GP-C40 was chosen to compare with OP-C40 for the SEM test in this study due to the identical characterizer of GP-C30, GP-C40 and GP-C50 after exposure to elevated temperature based on the XRD results. As shown in Figure 16, the morphology of unexposed GP-C40 was flat and dense, and contained some spherical particles, which are the unreacted fly ash. After exposure to 400 °C, the surface morphology of GP-C40 had no obvious change compared with that at room temperature. However, there were more cavities/pores in the cross-section morphology and the micro-structure became loose after 800 °C exposure, manifesting the serious degradation of specimen. Similar result showed that the average pore diameter and porosity of geopolymer paste (containing 25% slag and 75% fly ash) increased from 10.5 nm of 20 °C to 252.0 nm of 800 °C and from 24.08% of 20 °C to 34.43% of 800 °C, respectively [52]. As shown in Figure 17, the surface morphology of unexposed OP-C40 was also relatively flat and dense. Compared with unexposed GP-C40, OP-C40 has a certain honeycomb structure and acicular structure of C–S–H. After exposure to 400 °C, the morphology of OP-C40 is relatively flat and dense, and the needle like C–S–H has also no obvious change. The morphology of OP-C40 after 800 °C exposure was quite different from that of unexposed one, that is, obvious cracks appeared in the micro-structure, and the needle-like C–S–H was obviously reduced, indicating the hydrolysis/decomposition of C–S–H. The increased cracks were the result of thermal stresses that was generated due to the induced temperature gradients [55]. More similar SEM results of ordinary Portland cement based materials after elevated temperature were widely reported in [54,55,56,57]. Clearly, the effect of 400 °C on micro-structure of geopolymer and ordinary paste with grade of C40 is weak. Therefore, the effect of 400 °C on the mechanical properties of these two kinds of specimens is also small (See in Section 3.3). However, the effect of 800 °C on the micro-structure of geopolymer and ordinary paste was strong, especially for OP-C40 and obvious cracks were found, which caused a decrease in mechanical properties. SEM results also proved that C40 geopolymer paste is more resistant to elevated temperature than C40 ordinary paste.

## 4. Conclusions

The present study investigated the mechanical properties of fly ash and slag blend based geopolymer concrete, mortar and paste after elevated temperature exposure. Further, XRD, TGA and SEM were conducted to investigate the influences of mineralogical and micro-structural changes on the mechanical properties of geopolymer. Conclusions can be drawn from the experimental results and analysis presented in this paper.

(1) Different from ordinary concrete, mortar and paste, after 28 days, the compressive strength of geopolymer concrete, mortar and paste still increases significantly and the strengths of C40 geopolymer concrete, mortar and paste are significantly higher than that of C40 ordinary geopolymer concrete, mortar and paste.
(2) The compressive strength of C30, C40 and C50 geopolymer concrete, mortar and paste displays increment initially followed by a gradual reduction, and finally reached a relatively consistent value with the increase of exposure temperature, while the compressive strength of C40 ordinary concrete, mortar and paste remained approximately invariant before 400 °C, and then decreased rapidly. With the increase of concrete strength grade (i.e., the increase of slag content), the elevated temperature resistance of geopolymer concrete, mortar and paste gradually decreases. In addition, the elevated temperature resistance of C40 geopolymer concrete, mortar and paste is better than that of ordinary concrete, mortar and paste of the same grade.
(3) At the same heating temperature, GC-C30, GC-C40, GC-C50 and OC-C40 basically demonstrated the phenomenon that compressive strength retention has the best performance, which is followed by the splitting tensile strength, and flexural strength proved to be the lowest. The results indicated that the susceptibility of flexural strength, splitting tensile strength and compressive strength to elevated temperature decreased in turn for both geopolymer and ordinary concrete.
(4) XRD, TGA and SEM results showed that with the increase of concrete strength grade, the content of C–S–H formation in geopolymer increases, while the content of aluminosilicate gel, quartz and mullite decreases. Moreover, the heat resistance of C–S–H produced by slag is weaker than that of sulphoaluminate gel (quartz and mullite, etc.) produced by fly ash, which is the main reason for the decrease of C30, C40 and C50 geopolymer in turn after exposure to elevated temperatures.

## Figures and Tables

**Table 1:** XRF results of fly ash, slag and cement (Unit: %).

Oxide	SiO_2_	Al_2_O_3_	Fe_2_O_3_	CaO	K_2_O	SO_3_	TiO_2_	MgO	Na_2_O	P_2_O_5_	BaO	MnO	L.O.I ^a^
Fly ash	51.09	28.67	7.36	5.06	2.77	1.11	1.49	0.87	0.52	0.38	0.14	0.09	5.57
Slag	32.12	13.20	0.78	42.15	0.74	2.21	0.81	6.47	0.59	0.02	0.11	0.54	1.30
Cement	17.29	4.82	3.91	65.34	1.16	3.19	0.35	3.26	0.17	0.05	0.04	0.08	3.41

Note: ^a^: Loss on ignition.

**Table 2:** Details of concrete, mortar and paste mix proportions (mass per unit volume, kg).

Type	Binders			Sand	Coarse Aggregate		Alkaline Solutions		Water
	Fly Ash	Slag	Cement		5–10 mm	10–20 mm	SS ^a^	SH ^b^	
GC-C30	360	40	-	651	362.7	846.3	114.3	45.7	56.5
GC-C40	320	80	-	651	362.7	846.3	114.3	45.7	56.5
GC-C50	280	120	-	651	362.7	846.3	114.3	45.7	56.5
OC-C40	-	-	387	633	352.8	823.2	-	-	205
GM-C30	360	40	-	651	-		114.3	45.7	56.5
GM-C40	320	80	-	651	-		114.3	45.7	56.5
GM-C50	280	120	-	651	-		114.3	45.7	56.5
OM-C40	-	-	387	633	-		-	-	205
GP-C30	360	40	-	-	-		114.3	45.7	56.5
GP-C40	320	80	-	-	-		114.3	45.7	56.5
CP-C50	280	120	-	-	-		114.3	45.7	56.5
OP-C40	-	-	387	633	-		-	-	205

Note: ^a^: Sodium silicate solution; ^b^: Sodium hydroxide solution (with concentration of 14M); “-” means not available.

**Table 3:** Compressive strength of concrete, mortar and paste specimen with curing time.

Type	Compressive Strength (MPa)			
	3 Days	7 Days	28 Days	56 Days
GC-C30	10.1 (0.14)	13.5 (1.70)	31.9 (0.99)	37.0 (0.09)
GC-C40	17.7 (0.90)	25.6 (0.89)	40.4 (2.22)	48.2 (1.14)
GC-C50	27.8 (0.92)	35.0 (1.36)	48.0 (1.81)	58.8 (3.36)
OC-C40	24.8 (0.46)	31.3 (2.37)	39.2 (1.88)	41.5 (0.76)
GM-C30	7.3 (0.10)	12.3 (0.61)	25.8 (2.14)	39.7 (1.2)
GM-C40	14.5 (0.63)	20.0 (0.74)	40.0 (1.20)	46.7 (6.30)
GM-C50	22.7 (1.38)	33.2 (2.6)	52.3 (2.36)	65.9 (6.00)
OM-C40	21.1 (1.48)	25.5 (0.84)	39.2 (0.93)	40.0 (4.49)
GP-C30	7.5 (0.07)	12.5 (1.25)	27.8 (2.90)	46.8 (1.97)
GP-C40	15.0 (0.34)	23.4 (1.68)	34.4 (0.96)	56.5 (3.9)
CP-C50	26.2 (1.73)	32.2 (1.15)	50.7 (1.29)	59.6 (4.60)
OP-C40	18.7 (0.56)	21.9 (0.47)	30.3 (0.63)	33.2 (3.72)

Note: the values in parentheses are standard deviations.

**Table 4:** Tested compressive, splitting and flexural strength of concrete specimens.

Table.	Temperature ( °C)	Compressive Strength			Splitting Strength			Flexural Strength		
		Mean (MPa)	Retention (%)	COV ^a^ (%)	Mean (MPa)	Retention (%)	COV (%)	Mean (MPa)	Retention (%)	COV (%)
GC-C30	25	31.9	100	3.1	1.77	100	19.3	3.58	100	6.4
	100	38.5	120.8	1.7	3.05	172.8	20.5	4.57	127.8	11.8
	200	49.8	155.9	4.8	3.92	222.0	12.5	4.11	114.8	12.9
	300	51.6	161.7	5.0	2.18	123.6	0.5	2.91	81.2	5.3
	400	44.2	138.4	0.6	1.82	103.1	2.8	2.09	58.4	5.3
	500	43.1	135.1	10.3	1.23	69.4	2.6	1.68	46.9	2.5
	600	29.5	92.5	14.8	0.84	47.3	5.9	0.91	25.3	6.0
	700	17.3	54.2	6.9	0.44	24.8	2.0	0.56	15.6	1.6
	800	17.0	53.3	7.4	0.40	22.6	2.0	0.40	11.1	2.4
GC-C40	25	40.4	100	5.5	2.45	100	16.6	4.15	100	17.6
	100	51.0	126.0	2.6	3.12	127.1	1.4	4.53	109.1	13.4
	200	58.1	143.6	4.7	3.32	135.5	12.6	3.22	77.5	7.5
	300	53.7	132.8	18.7	2.15	87.6	7.5	2.26	54.4	4.8
	400	49.5	122.3	5.2	1.51	61.6	5.3	1.66	39.9	1.9
	500	43.5	107.7	9.0	1.19	48.5	8.5	0.98	23.5	2.5
	600	26.7	66.1	8.9	0.71	28.8	1.0	0.47	11.2	0.3
	700	16.0	39.5	3.5	0.38	15.5	0.6	0.38	9.1	0.7
	800	14.1	35.1	4.2	0.40	16.3	1.4	0.26	6.3	0.4
GC-C50	25	48.0	100	3.8	3.01	100	4.9	4.77	100	15.4
	100	60.5	126.0	7.2	4.28	142.1	13.3	4.71	98.8	4.6
	200	60.0	124.9	12.8	3.14	104.2	7.4	3.07	64.3	4.1
	300	52.9	110.1	2.8	2.12	70.3	3.3	2.51	52.6	1.0
	400	53.0	110.3	5.4	1.64	54.3	6.8	1.96	41.0	4.7
	500	41.0	85.4	5.9	1.35	44.8	10.1	1.28	26.9	1.2
	600	27.8	57.8	9.5	0.78	25.8	3.6	0.64	13.5	3.3
	700	12.5	26.0	0.8	0.36	12.0	1.8	0.33	7.0	0.3
	800	12.1	25.2	0.6	0.36	12.1	1.3	0.23	4.9	0.8
OC-C40	25	39.2	100	1.4	2.51	100	10.4	3.95	100	7.3
	100	36.8	93.8	2.8	2.30	91.6	3.8	3.70	93.7	6.1
	200	34.8	88.7	5.7	1.86	74.3	8.3	2.91	73.8	6.9
	300	39.8	101.5	7.4	1.74	69.5	9.1	2.20	55.7	8.7
	400	37.3	95.0	7.1	0.94	37.6	2.5	0.98	24.7	1.3
	500	34.1	86.8	2.7	0.89	35.4	1.5	0.97	24.6	5.0
	600	19.5	49.7	10.1	0.42	16.6	3.8	0.52	13.0	0.8
	700	14.2	36.3	3.3	0.32	12.8	2.5	0.20	5.2	0.8
	800	7.2	18.4	2.1	0.24	9.7	1.3	0.10	2.4	1.4

Note: ^a^: Coefficient of variation.

**Table 5:** Tested compressive strength of mortar and paste specimens.

Type	Temperature (°C)	Mortar			Paste		
		Mean (MPa)	Retention (%)	COV ^a^ (%)	Mean (MPa)	Retention (%)	COV (%)
GM-C30 or GP-C30	25	25.8	100	8.3	27.8	100	10.4
	100	41.1	159.6	12.0	42.7	153.5	9.1
	200	65.8	255.2	6.0	45.2	162.7	17.0
	300	53.1	206.0	14.0	51.9	186.9	19.6
	400	45.0	174.4	7.2	33.4	120.4	16.2
	500	42.3	163.9	5.6	28.1	101.2	3.6
	600	22.3	86.5	2.4	15.8	57.0	5.2
	700	19.4	75.4	0.6	17.3	62.1	5.0
	800	21.0	81.6	2.3	18.2	65.6	7.5
GM-C40 or GP-C40	25	40.0	100	3.0	34.4	100	12.7
	100	55.1	137.5	10.9	45.7	132.6	16.5
	200	58.1	145.1	10.2	39.3	114.2	11.3
	300	49.9	124.6	8.6	41.1	119.4	17.5
	400	42.1	105.1	3.5	26.8	77.9	8.5
	500	36.3	90.7	2.9	17.2	50.1	3.9
	600	18.2	45.5	1.7	9.3	27.0	2.1
	700	19.0	47.5	2.4	11.6	33.7	2.6
	800	21.7	54.3	0.8	16.7	48.5	3.4
GM-C50 or GP-C50	25	52.3	100	4.5	50.7	100	2.5
	100	63.8	122.0	7.6	49.5	97.6	6.9
	200	71.0	135.8	2.3	42.1	83.0	5.8
	300	59.3	113.4	5.3	57.0	112.3	7.7
	400	49.8	95.3	4.5	38.5	76.0	10.6
	500	45.2	86.5	5.8	31.0	61.0	9.6
	600	20.0	38.2	2.9	9.5	18.7	1.9
	700	18.9	36.2	2.1	15.8	31.2	6.5
	800	24.3	46.4	1.7	15.8	31.2	1.5
OM-C40 or OP-C40	25	39.2	100	2.4	30.3	100	3.0
	100	39.0	99.7	1.0	29.1	96.0	7.1
	200	34.7	88.5	12.0	24.5	80.9	7.1
	300	38.9	99.4	8.1	31.5	103.9	6.6
	400	29.4	75.1	7.2	24.8	81.7	10.3
	500	29.5	75.4	7.8	13.7	45.1	6.9
	600	13.2	33.7	2.3	5.6	18.5	4.4
	700	9.4	24.0	3.2	6.3	20.6	2.3
	800	6.4	16.3	0.6	3.8	12.4	1.9

Note: ^a:^ Coefficient of variation.

## Data Availability

The data presented in this study are available on request from the corresponding author.

## References

[1] Kong D.L., Sanjayan J.G. (2008). Damage behavior of geopolymer composites exposed to elevated temperatures. Cem. Concr. Compos..

[2] I.E. Agency (2020). Global Cement Production, 2010–2019. https://www.iea.org/data-and-statistics/charts/global-cement-production-2010-2019.

[3] Van Deventer J.S., Provis J.L., Duxson P. (2012). Technical and commercial progress in the adoption of geopolymer cement. Miner. Eng..

[4] Assi L.N., Deaver E., ElBatanouny M.K., Ziehl P. (2016). Investigation of early compressive strength of fly ash-based geopolymer concrete. Constr. Build. Mater..

[5] Visintin P., Ali M.M., Albitar M., Lucas W. (2017). Shear behaviour of geopolymer concrete beams without stirrups. Constr. Build. Mater..

[6] Chen C., Habert G., Bouzidi Y., Jullien A. (2010). Environmental impact of cement production: Detail of the different processes and cement plant variability evaluation. J. Clean. Prod..

[7] Davidovits J. (1991). Geopolymers. J. Therm. Anal. Calorim..

[8] Hardjito D., Wallah S.E., Sumajouw D.M.J., Rangan B.V. (2004). On the development of fly ash-based geopolymer concrete. ACI Mater. J..

[9] Kong D.L., Sanjayan J.G., Sagoe-Crentsil K. (2007). Comparative performance of geopolymers made with metakaolin and fly ash after exposure to elevated temperatures. Cem. Concr. Res..

[10] Zhang H.Y., Kodur V., Wu B., Cao L., Wang F. (2016). Thermal behavior and mechanical properties of geopolymer mortar after exposure to elevated temperatures. Constr. Build. Mater..

[11] Bakharev T., Sanjayan J.G., Cheng Y.-B. (1999). Alkali activation of Australian slag cements. Cem. Concr. Res..

[12] Sarker P.K., Mcbeath S. (2015). Fire endurance of steel reinforced fly ash geopolymer concrete elements. Constr. Build. Mater..

[13] Duxson P., Fernández-Jiménez A., Provis J.L., Lukey G.C., Palomo Á., Van Deventer J.S.J. (2007). Geopolymer technology: The current state of the art. J. Mater. Sci..

[14] Kong D.L., Sanjayan J.G. (2010). Effect of elevated temperatures on geopolymer paste, mortar and concrete. Cem. Concr. Res..

[15] Ali A.M., Sanjayan J., Guerrieri M. (2017). Performance of geopolymer high strength concrete wall panels and cylinders when exposed to a hydrocarbon fire. Constr. Build. Mater..

[16] Zhang H.Y., Kodur V., Wu B., Cao L., Qi S.L. (2016). Comparative Thermal and Mechanical Performance of Geopolymers derived from Metakaolin and Fly Ash. J. Mater. Civ. Eng..

[17] Chowdhury S., Mohapatra S., Gaur A., Dwivedi G., Soni A. (2020). Study of various properties of geopolymer concrete—A review. Mater. Today Proc..

[18] Colangelo F., Cioffi R., Roviello G., Capasso I., Caputo D., Aprea P., Liguori B., Ferone C. (2017). Thermal cycling stability of fly ash based geopolymer mortars. Compos. Part B Eng..

[19] Li C., Xian G., Li H. (2018). Influence of immersion in water under hydraulic pressure on the interfacial shear strength of a unidirectional carbon/glass hybrid rod. Polym. Test..

[20] Pan Z., Tao Z., Cao Y., Wuhrer R., Murphy T. (2018). Compressive strength and microstructure of alkali-activated fly ash/slag binders at high temperature. Cem. Concr. Compos..

[21] Sarker P.K., Kelly S., Yao Z. (2014). Effect of fire exposure on cracking, spalling and residual strength of fly ash geopolymer concrete. Mater. Des..

[22] Ranjbar N., Mehrali M., Alengaram U.J., Simon H., Jumaat Z. (2014). Compressive strength and microstructural analysis of fly ash/palm oil fuel ash based geopolymer mortar under elevated temperatures. Mater. Des..

[23] Abdulkareem O.A., Al Bakri A.M., Kamarudin H., Nizar I.K., Saif A.A. (2014). Effects of elevated temperatures on the thermal behavior and mechanical performance of fly ash geopolymer paste, mortar and lightweight concrete. Constr. Build. Mater..

[24] Fernández-Jiménez A.M., Palomo A., López-Hombrados C. (2006). Engineering properties of alkali-activated fly ash. ACI Mater. J..

[25] Nath P., Sarker P.K. (2015). Use of OPC to improve setting and early strength properties of low calcium fly ash geopolymer concrete cured at room temperature. Cem. Concr. Compos..

[26] Lee N., Lee H. (2013). Setting and mechanical properties of alkali-activated fly ash/slag concrete manufactured at room temperature. Constr. Build. Mater..

[27] Nath P., Sarker P.K. (2014). Effect of GGBFS on setting, workability and early strength properties of fly ash geopolymer concrete cured in ambient condition. Constr. Build. Mater..

[28] Al-Majidi M.H., Lampropoulos A., Cundy A., Meikle S. (2016). Development of geopolymer mortar under ambient temperature for in situ applications. Constr. Build. Mater..

[29] Nath P., Sarker P.K. (2016). Fracture properties of GGBFS-blended fly ash geopolymer concrete cured in ambient temperature. Mater. Struct..

[30] Fang G., Ho W.K., Tu W., Zhang M. (2018). Workability and mechanical properties of alkali-activated fly ash-slag concrete cured at ambient temperature. Constr. Build. Mater..

[31] Lee N., Koh K., An G., Ryu G. (2017). Influence of binder composition on the gel structure in alkali activated fly ash/slag pastes exposed to elevated temperatures. Ceram. Int..

[32] Park S., Jang J., Lee N., Lee H. (2016). Physicochemical properties of binder gel in alkali-activated fly ash/slag exposed to high temperatures. Cem. Concr. Res..

[33] Qu F., Li W., Tao Z., Castel A., Wang K. (2020). High temperature resistance of fly ash/GGBFS-based geopolymer mortar with load-induced damage. Mater. Struct..

[34] Wang Y.C., Wong P.M.H., Kodur V. (2007). An experimental study of the mechanical properties of fibre reinforced polymer (FRP) and steel reinforcing bars at elevated temperatures. Compos. Struct..

[35] Wang S., Vipulanandan C. (2000). Solidification/stabilization of Cr(VI) with cement leachability and XRD analyses. Cem. Concr. Res..

[36] Deb P.S., Nath P., Sarker P.K. (2014). The effects of ground granulated blast-furnace slag blending with fly ash and activator content on the workability and strength properties of geopolymer concrete cured at ambient temperature. Mater. Des..

[37] (2019). G.T. 50081-2019. Standard for Test Methods of Concrete Physical Physical and Mechanical Properties.

[38] (2009). JGJ/T70-2009. Standard for Test Method of Performance on Building Mortar.

[39] Pan Z., Tao Z., Cao Y.-F., Wuhrer R. (2018). Measurement and prediction of thermal properties of alkali-activated fly ash/slag binders at elevated temperatures. Mater. Struct..

[40] Kumar S., Kumar R., Mehrotra S.P. (2010). Influence of granulated blast furnace slag on the reaction, structure and properties of fly ash based geopolymer. J. Mater. Sci..

[41] Lim S., Mondal P. (2014). Micro- and nano-scale characterization to study the thermal degradation of cement-based materials. Mater. Charact..

[42] Alonso C., Fernandez L. (2004). Dehydration and rehydration processes of cement paste exposed to high temperature environments. J. Mater. Sci..

[43] Komonen J., Penttala V. (2003). Effects of High Temperature on the Pore Structure and Strength of Plain and Polypropylene Fiber Reinforced Cement Pastes. Fire Technol..

[44] Škvára F., Kopecký L., Šmilauer V., Bittnar Z. (2009). Material and structural characterization of alkali activated low-calcium brown coal fly ash. J. Hazard. Mater..

[45] Peng G.-F., Huang Z.-S. (2008). Change in microstructure of hardened cement paste subjected to elevated temperatures. Constr. Build. Mater..

[46] Xiao J., König G. (2004). Study on concrete at high temperature in China—an overview. Fire Saf. J..

[47] Zhao Z., Qu X., Li J. (2020). Microstructure and properties of fly ash/cement-based pastes activated with MgO and CaO under hydrothermal conditions. Cem. Concr. Compos..

[48] Zhao X., Liu C., Wang L., Zuo L., Zhu Q., Ma W. (2019). Physical and mechanical properties and micro characteristics of fly ash-based geopolymers incorporating soda residue. Cem. Concr. Compos..

[49] Li C. (2020). Mechanical and transport properties of recycled aggregate concrete modified with limestone powder. Compos. Part B Eng..

[50] Bernal S.A., Provis J.L., Walkley B., Nicolas R.S., Gehman J.D., Brice D.G., Kilcullen A.R., Duxson P., Van Deventer J.S. (2013). Gel nanostructure in alkali-activated binders based on slag and fly ash, and effects of accelerated carbonation. Cem. Concr. Res..

[51] Kürklü G. (2016). The effect of high temperature on the design of blast furnace slag and coarse fly ash-based geopolymer mortar. Compos. Part B Eng..

[52] Lee N.K., Koh K.T., Kim M.O., An G.H., Ryu G.S. (2017). Physicochemical changes caused by reactive MgO in alkali-activated fly ash/slag blends under accelerated carbonation. Ceram. Int..

[53] Castellote M., Alonso C., Andrade C., Turrillas X., Campo J. (2004). Composition and microstructural changes of cement pastes upon heating, as studied by neutron diffraction. Cem. Concr. Res..

[54] Saridemir M., Severcan M., Ciflikli M., Celikten S., Ozcan F., Atis C. (2016). The influence of elevated temperature on strength and microstructure of high strength concrete containing ground pumice and metakaolin. Constr. Build. Mater..

[55] Hussin M.W., Bhutta M.A.R., Azreen M., Ramadhansyah P.J., Mirza J. (2014). Performance of blended ash geopolymer concrete at elevated temperatures. Mater. Struct..

[56] Demirel B., Keleştemur O. (2010). Effect of elevated temperature on the mechanical properties of concrete produced with finely ground pumice and silica fume. Fire Saf. J..

[57] Akca A.H., Zihnioğlu N. (2013). Özyurt High performance concrete under elevated temperatures. Constr. Build. Mater..

